# Managing Cancer and Living Meaningfully (CALM): A Randomized Controlled Trial of a Psychological Intervention for Patients With Advanced Cancer

**DOI:** 10.1200/JCO.2017.77.1097

**Published:** 2018-06-29

**Authors:** Gary Rodin, Christopher Lo, Anne Rydall, Joanna Shnall, Carmine Malfitano, Aubrey Chiu, Tania Panday, Sarah Watt, Ekaterina An, Rinat Nissim, Madeline Li, Camilla Zimmermann, Sarah Hales

**Affiliations:** All authors: Princess Margaret Cancer Centre; Gary Rodin, Christopher Lo, Rinat Nissim, Madeline Li, Camilla Zimmermann, and Sarah Hales, University of Toronto; Toronto, Ontario, Canada.

## Abstract

**Purpose:**

Individuals with advanced cancer experience substantial distress in response to disease burden and impending mortality. Managing Cancer And Living Meaningfully (CALM) is a novel, brief, manualized psychotherapeutic intervention intended to treat and prevent depression and end-of-life distress in patients with advanced cancer. We conducted a randomized controlled trial to compare CALM with usual care (UC) in this population.

**Methods:**

Patients with advanced cancer were recruited from outpatient oncology clinics at a comprehensive cancer center into an unblinded randomized controlled trial. Permuted block randomization stratified by Patient Health Questionnaire-9 depression score allocated participants to CALM plus UC or to UC alone. Assessments of depressive symptoms (primary outcome), death-related distress, and other secondary outcomes were conducted at baseline, 3 months (primary end point), and 6 months (trial end point). Analyses were by intention to treat. Analysis of covariance was used to test for outcome differences between groups at follow-up, controlling for baseline. Mixed-model results are reported.

**Results:**

Participants (n = 305) were recruited between February 3, 2012, and March 4, 2016, and randomly assigned to CALM (n = 151) or UC (n = 154). CALM participants reported less-severe depressive symptoms than UC participants at 3 months (Δ = 1.09; *P* = .04; Cohen’s *d* = 0.23; 95% CI, 0.04 to 2.13) and at 6 months (Δ = 1.29; *P* = .02; *d* = 0.29; 95% CI, 0.24 to 2.35). Significant findings for greater end-of-life preparation at 6 months also favored CALM versus UC. No adverse effects were identified.

**Conclusion:**

Findings suggest that CALM is an effective intervention that provides a systematic approach to alleviating depressive symptoms in patients with advanced cancer and addresses the predictable challenges these patients face.

## INTRODUCTION

The diagnosis of advanced cancer may trigger enormous distress and the challenge of living meaningfully in the face of progressive disease. Individuals in this situation face the burden of physical suffering, the threat of dependency and impending mortality, and the difficulty of making treatment decisions that have life-and-death implications while navigating a complex health care system.^1^ Early palliative care for such individuals has been shown to produce better outcomes,^2-4^ but the psychological dimensions of such care are much less systematized than those focused on symptom control and advance care planning.

Ground-breaking research on supportive-expressive therapy has demonstrated positive effects on psychological outcomes in women with metastatic breast cancer.^5-7^ More recently, three systematic reviews of randomized controlled trials (RCTs) confirmed that psychotherapy is effective in treating depressive states in individuals with advanced cancer, despite methodological limitations in most studies.^8-10^ Both Dignity Therapy,^11^ a legacy-building intervention for those near the very end of life, and Meaning-Centered Psychotherapy,^12,13^ a group or individual intervention that promotes a sense of meaning and purpose in patients with advanced cancer, have been shown to be effective in a variety of outcomes.^13-15^

We have developed a novel, brief, tailored supportive-expressive psychotherapeutic intervention, referred to as Managing Cancer And Living Meaningfully (CALM) for patients with advanced cancer and a prognosis of at least 1 year.^16^ On the basis of relational, attachment, and existential theory, CALM provides a therapeutic relationship and reflective space, with attention to the following domains: symptom management and communication with health care providers, changes in self and relations with close others, spiritual well-being and the sense of meaning and purpose, and mortality and future-oriented concerns.^17^ The CALM domains are addressed for each patient in a tailored, individualized manner that allows for variation in the number of sessions and time spent on each domain on the basis of the patient’s needs and health status. CALM can be delivered by a wide range of trained psychosocial oncology clinicians and cancer care providers.^17^

In pilot trials with patients with advanced cancer, we demonstrated that CALM is feasible and found evidence of improvement in depression, death anxiety, spiritual well-being, and attachment security.^18,19^ In qualitative interviews, participants reported that CALM provides a “safe place” that helped them to “be seen as a whole person by the medical system,” “grow as a person,” and “be able to handle death in a peaceful way.”^20^ We now report quantitative findings from an RCT of CALM. The primary outcome was the severity of depressive symptoms, which was selected because of evidence that depression is a final common pathway of distress in this population.^21^ The primary end point of 3 months was chosen a priori to minimize the effects of attrition as a result of disease progression; the secondary end point was 6 months. Secondary outcomes were selected on the basis of the theoretical underpinnings of CALM and prior research^21,22^ and included diagnosis of major depression, generalized anxiety, death-related anxiety, spiritual well-being, quality of life at the end of life, attachment security, couple communication, post-traumatic growth, and demoralization.

## METHODS

### Study Design

This unblinded, parallel assignment RCT had two trial conditions: intervention plus usual care (UC) versus UC alone, with assessments at baseline (t0), 3 months (t1; primary end point), and 6 months (t2; trial end point). The trial protocol is provided in Lo et al.^23^ The site was the Princess Margaret Cancer Centre (PM), which is part of the University Health Network in Toronto, Ontario, Canada. This trial was approved by the University Health Network Research Ethics Board (REB #09-0855-C) and registered with ClinicalTrials.gov.

### Participants

Inclusion criteria were ≥ 18 years of age; English fluency; no cognitive impairment; and diagnosis of stage III or IV lung cancer, any-stage pancreatic cancer (because of its aggressiveness), unresectable cholangiocarcinoma, unresectable liver cancer, unresectable ampullary or peri-ampullary cancer or other stage IV gastrointestinal (GI) cancer, stage III or IV ovarian and fallopian tube cancers or other stage IV gynecologic cancer, stage IV breast cancer, genitourinary cancer, sarcoma, melanoma, or endocrine cancer. Diagnoses were confirmed by chart review and consistent with an expected prognosis of 12 to 18 months on the basis of prior research in this population.^22^ Exclusion criteria were major communication difficulties, cognitive impairment on the basis of a Short Orientation-Memory-Concentration test score < 20,^24^ current psychiatric or psychological treatment in the Department of Supportive Care at PM, unwillingness to accept random assignment or to commit to the study, and prior participation in CALM therapy. Participants were approved for trial enrollment by the principal investigators before random assignment and provided written informed consent.

### Randomization and Masking

Permuted block randomization was used to allocate participants, with stratification by Patient Health Questionnaire-9 (PHQ-9) score (< 10 or ≥ 10)^25^ to ensure balance of moderate to severe depressive symptoms between arms. The PM Biostatistics Department, which is independent of the trial team, managed the randomization. Block sizes were variable and unknown to the research team. Computer-generated randomization assignments were provided by the Biostatistics Department after the participant’s baseline assessment.

### Trial Conditions

On the basis of earlier trials,^18,19^ most intervention participants were expected to receive three to six CALM psychotherapy sessions (each 45 to 60 minutes) over 3 to 6 months. The actual number of sessions each participant received was based on clinical judgment and the patient’s ability to participate. Therapists aimed to deliver at least three sessions within the first 3 months of study enrollment. Noncompliance was defined as fewer than three sessions over the course of the trial. The primary caregiver was invited to one or more sessions when acceptable to the participant and therapist. Therapists were five master’s degree–level social workers and three psychiatrists. CALM training involved a 2-day workshop and satisfactory completion of at least two cases under supervision with G.R. and S.H., codevelopers of the intervention.^16^ Treatment integrity was maintained through weekly group supervision, which included a review of session audiotapes and case discussion. After case presentations, G.R. and S.H. used a treatment integrity rating scale (Appendix, online only) adapted from Spiegel and Spira^26^ to assess the therapists, and these evaluations were discussed to improve competencies.

Participants in the control group received UC alone (see Table 1 for comparison with CALM), which included routine oncology treatment and follow-up and clinic-based distress screening.^27-29^ UC did not preclude referral for specialized psychosocial oncology services, but most patients with metastatic cancer at PM do not receive psychotherapy as part of UC.^30^

**Table 1.**
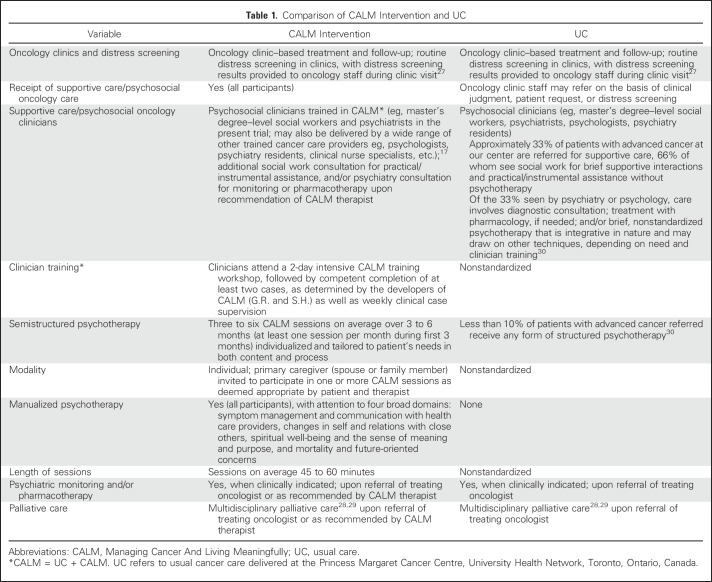
Comparison of CALM Intervention and UC

### Procedures

Patients with advanced cancer were identified through prescreening of outpatient oncology clinic lists, and eligible patients were approached for recruitment during clinic appointments. After informed consent, research staff assessed cognitive functioning, readiness, and ability to participate, administered baseline measures, conducted a diagnostic interview for depression, and received enrollment approval from the study principal investigators before contacting the PM Biostatistics Department to receive randomization assignments. Participants were contacted at 3 and 6 months to complete follow-up assessments, which were conducted in person at the hospital, by telephone, or by mail. Assessments were scanned and scored by an independent data management company. The final data set was exported to the trial team and to a biostatistician at PM (not part of the trial team) for analysis and verification.

### Outcome Measures

#### Primary outcome.

The primary outcome was measured using the PHQ-9,^25^ a reliable and valid measure of Diagnostic and Statistical Manual of Mental Disorders, Fourth Edition (DSM-IV)^31^–concordant depression. The PHQ-9 has been widely used in patients with advanced cancer.^32^

#### Secondary outcomes.

The secondary outcomes were measured with the following assessments:

Structured Clinical Interview for DSM-IV-TR Axis I Disorders (SCID), Research Version,^33^ a semistructured interview for the diagnosis of DSM-IV major depression^31^ (administered by research staff trained and supervised by M.L.)
Generalized Anxiety Disorder-7 (GAD-7),^34^ a widely used and validated measure to assess generalized anxiety symptoms
Death and Dying Distress Scale (DADDS),^35^ a valid measure in patients with advanced cancer^36^ that rates distress about the dying process, lost opportunities, and perceived burden on others
Functional Assessment of Chronic Illness Therapy-Spiritual Well-Being Scale (FACIT-Sp-12),^37^ a measure of spiritual well-being validated for use in cancer^38^ and widely used in palliative care research^39^
Quality of Life at the End of Life Cancer Scale (QUAL-EC),^40^ a short form of the Quality of Life at the End of Life assessment^41^; we used the following subscales relevant to psychosocial functioning: preparation for the end of life (ie, extent to which the family is prepared and financial plans made), relationship with health care providers (ie, extent to which patients feel informed and are able to participate in decisions about their care), and sense of life completion (ie, ability to share important things and to feel connected to others)
16-Item Experiences in Close Relationships Scale validated for use in advanced cancer (ECR-M16),^42^ a modified and brief version of the Experiences in Close Relationships assessment^43^ that measures attachment insecurity (ie, reflects difficulty in trusting and relying on close others in times of need)^44^
Couple Communication Scale (CCS)^45^ for participants in long-term relationships, a part of the validated PREPARE/ENRICH Inventory^45^ that assesses the quality of communication in the dyad
Posttraumatic Growth Inventory (PTGI),^46^ a valid measure previously used in cancer to assess positive psychological changes after trauma^47^
Demoralization Scale (DS),^48^ a validated measure of the experience of disheartenment and helplessness

Additional data collected were demographics, medical history, Karnofsky performance status,^49^ and the presence and severity of 28 common cancer symptoms assessed using a shortened version of the Memorial Symptom Assessment Scale.^50^

### Statistical Analysis

A sample size recalculation was conducted in February 2014 using actual attrition and compliance rates rather than pretrial estimates and without examination of treatment effects. A total baseline sample of at least 242 participants would power this trial to detect a small to medium effect size (Cohen’s *d*)^51^ of 0.405.^19^ With available resources, we chose to extend recruitment to reach at least 100 trial completionists per arm, which was achieved after consenting 413 participants, 305 of whom were randomly assigned.

Analyses were by intention to treat. Analysis of covariance was used to examine outcome differences between trial arms at follow-up, controlling for baseline scores. The main analyses were conducted on available participants, *P* values correspond to two-tailed tests and α was set at .05. As a sensitivity analysis, we used multiple imputation with the Markov model^52^ to address the issue of missing data (Appendix Table A1, online only) and report *P* values that tested the aggregate results of 20 imputations, which achieved 0.99 relative efficiency on the primary outcome and stabilized estimates. The imputation model included the relevant t0, t1, and t2 outcome assessments and randomization. We used the false discovery rate (FDR)^53^ method to control for multiple comparisons on the secondary outcomes and report FDR-adjusted *P* values. The FDR was applied separately to the family of tests at 3 and 6 months. The familywise FDR was set to .05. Trial analyses were independently verified by a member of the PM Biostatistics Department. Analyses for this article were generated using SAS/STAT 9.3 statistical software (SAS Institute, Cary, NC).

To clarify the clinical meaning of effects on the primary outcome, we conducted post hoc analyses with regard to the emergence and remission rates of depressive symptoms of at least threshold severity (indicated by PHQ-9 ≥ 8 points),^54^ and the proportion of patients with a PHQ-9 reduction greater than the minimal clinically important difference (MCID) of 5 points^55^ in participants with depressive symptoms of at least threshold severity. Prespecified subanalyses were conducted for groups with low, moderate, or high death anxiety at baseline (using DADDS cutoffs of < 25 and ≥ 47 points to distinguish approximately the upper and lower thirds of the sample) because these groups may differ in the processing of death-related distress.^56^ Finally, mixed models were conducted as supplementary analyses to examine treatment effects across outcomes, regressing each outcome on trial arm (UC, CALM), time (t0, t1, t2), and their interaction, with intercepts set as random effects.

## RESULTS

**Fig 1.**
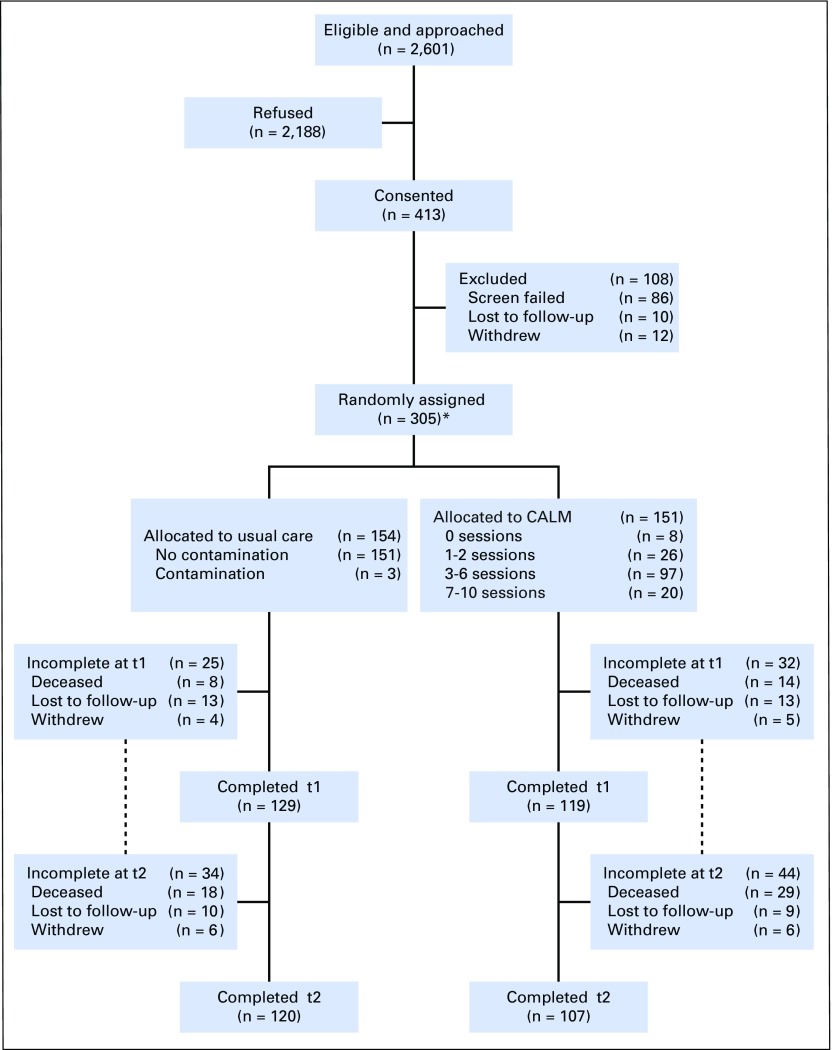
CONSORT diagram. (*) Analyses by intention to treat. CALM, Managing Cancer And Living Meaningfully.

**Fig A1.**
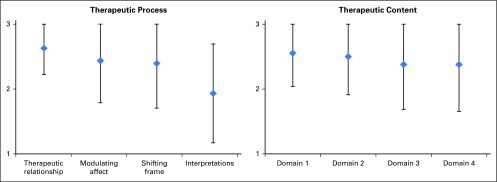
Treatment integrity ratings (n = 51). Values on the y-axis indicate the following ratings: 1 = needs improvement, 2 = satisfactory, 3 = excellent. The therapeutic relationship refers to the therapist’s ability to support patient reflection within an empathic relationship. Modulating affect refers to being able to assist patients in managing negative emotions. Shifting frame refers to being able to adjust one’s clinical approach on the basis of patient needs. Interpretations refer to the ability to offer psychological explanations to foster dialogue with the patient. Domain 1, symptom management and communication with health care providers; domain 2, changes in self and relations with close others; domain 3, spirituality or sense of meaning and purpose; domain 4, thinking of the future, hope, and mortality. See the Treatment Integrity Scale: Managing Cancer And Living Meaningfully Evaluation of Therapist Competencies.

Four hundred and thirteen patients consented to participate between February 3, 2012, and March 4, 2016, 305 of whom were randomly assigned to CALM (n = 151) and UC (n = 154; Fig 1). Contamination (defined as two or more psychotherapy sessions with a CALM-trained PM clinician) was 2% in control participants. Participation in at least three sessions was considered a minimal intervention. On the basis of this criterion, compliance with the intervention was 54.3% in the CALM group at 3 months (mean, 3 sessions; range, 0 to 7 sessions) and 77.5% by 6 months (mean, 4 sessions; range, 0 to 10 sessions). Of those who received three or more sessions over 6 months, 64.2% received three to six sessions and 13.3% received seven to 10 sessions; of the remaining CALM participants, 17.2% received one or two sessions and 5.3% received no sessions. The majority of sessions were delivered in outpatient clinics; a small number were delivered to very ill participants by telephone or in the inpatient palliative care unit. Mean treatment integrity ratings indicated that most therapeutic competencies were satisfactory to excellent, with the most room for improvement in the offering of interpretations (Appendix Fig A1, online only). The overall attrition rate at 6 months (trial end point) was 25.5% (15.4% died, 6.2% were lost to follow-up, and 3.9% withdrew), with 70.9% of the CALM group and 77.9% of the UC group completing the trial. Final trial follow-ups were completed by September 2016. Mortality 1 year after trial completion was 67.5% (206 of 305 participants [109 CALM participants, 97 UC participants]).

No trial group differences existed at baseline (Table 2), except for antidepressant use (18% UC *v* 10% CALM). Preliminary analyses that controlled for this factor found that it was nonsignificant and did not affect the pattern and magnitude of findings; therefore, we report group differences without covariables. Of note, as the study progressed, the group difference on antidepressant use was nonsignificant at 3 months (UC, 19.5% [25 of 128]; CALM, 11.8% [14 of 119]; *P* = .09) and at 6 months (UC, 17.1% [20 of 117]; CALM, 12.3% [13 of 106]; *P* = .31).

**Table 2.**
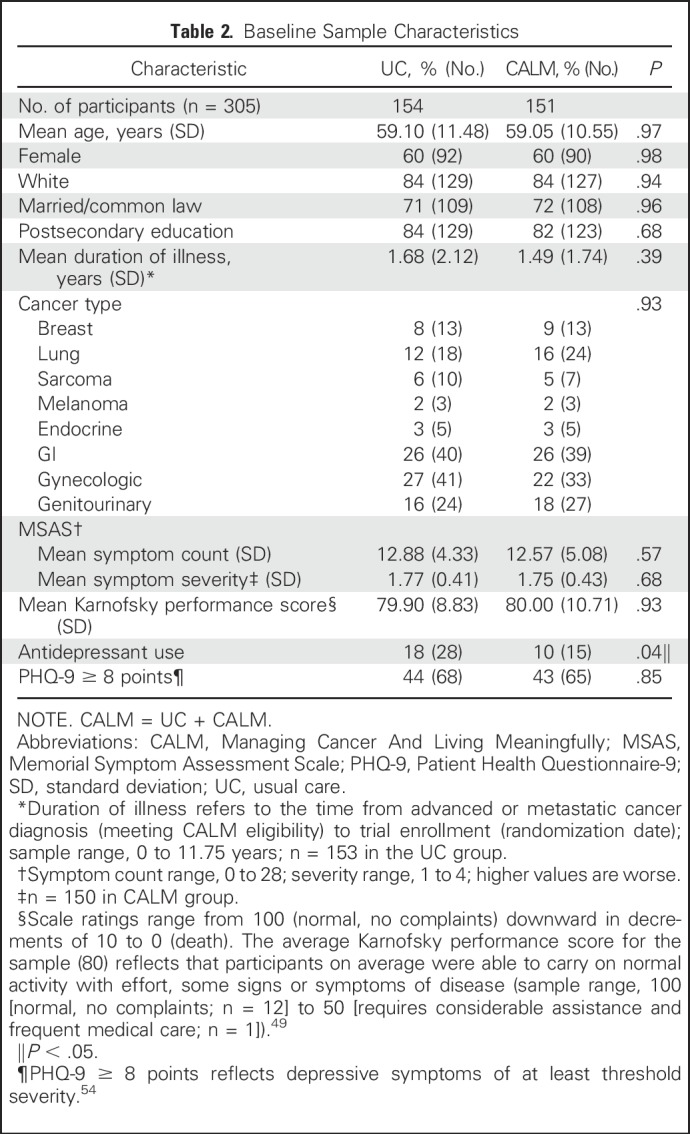
Baseline Sample Characteristics

Table 3 lists primary outcome results. The CALM group reported less-severe depressive symptoms than the UC group at the primary end point of 3 months (*d* = 0.23; *P* = .04). This effect appeared to be greater at 6 months (*d* = 0.29; *P* = .02). Analysis of multiple imputations yielded the same patterns of effect.

**Table 3.**
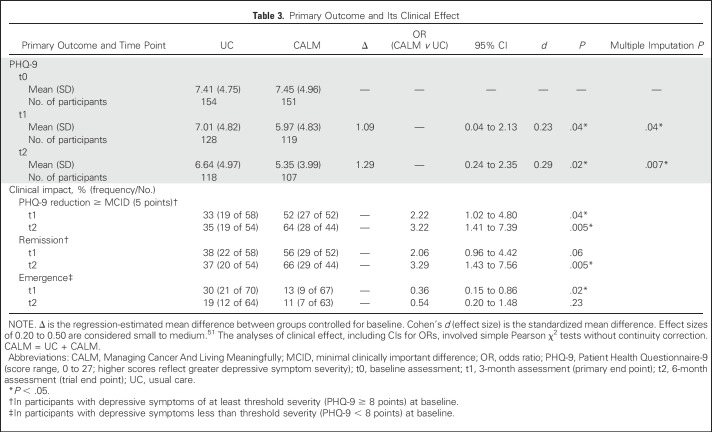
Primary Outcome and Its Clinical Effect

To clarify clinical meaning, we conducted post hoc analyses of remission and emergence rates of at least threshold depression (PHQ-9 ≥ 8 points^54^; Table 3). CALM participants were more likely to show remission of symptoms of at least threshold severity at 6 months (odds ratio [OR], 3.29; *P* = .005) and were less likely to develop depressive symptoms of at least threshold severity at 3 months (OR, 0.36; *P* = .02). For participants with depressive symptoms of at least threshold severity, CALM was more likely to provide a clinically important PHQ-9 reduction (minimal clinically important difference [MCID], 5 points)^55^ at 3 months (OR, 2.22; *P* = .04) and at 6 months (OR, 3.22; *P* = .005).

Table 4 lists the secondary outcomes, and Table 5 lists the FDR-adjusted *P* values and multiple imputation results. With a focus on the most robust findings, a significant treatment effect was found for preparation for end of life at both 3 and 6 months in CALM participants compared with UC that was sustained after multiple imputation, although the 3-month effect was rendered nonsignificant after controlling for multiple comparisons. No adverse effects were reported. Some outcomes (couple communication and relationship with health care providers) were better in the UC group at 3 months, although these effects were rendered nonsignificant after multiple imputations and controlling for multiple comparisons and were not sustained at 6 months.

**Table 4.**
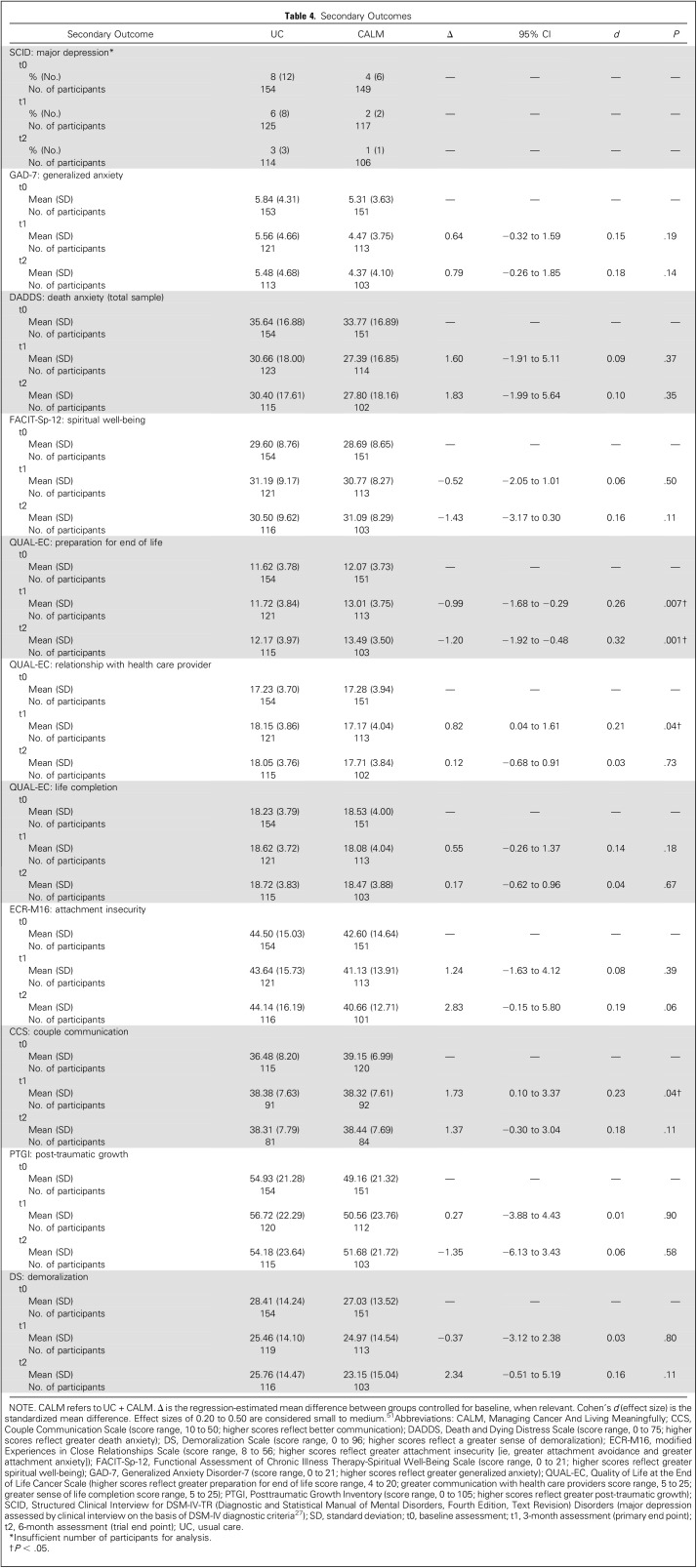
Secondary Outcomes

**Table 5.**
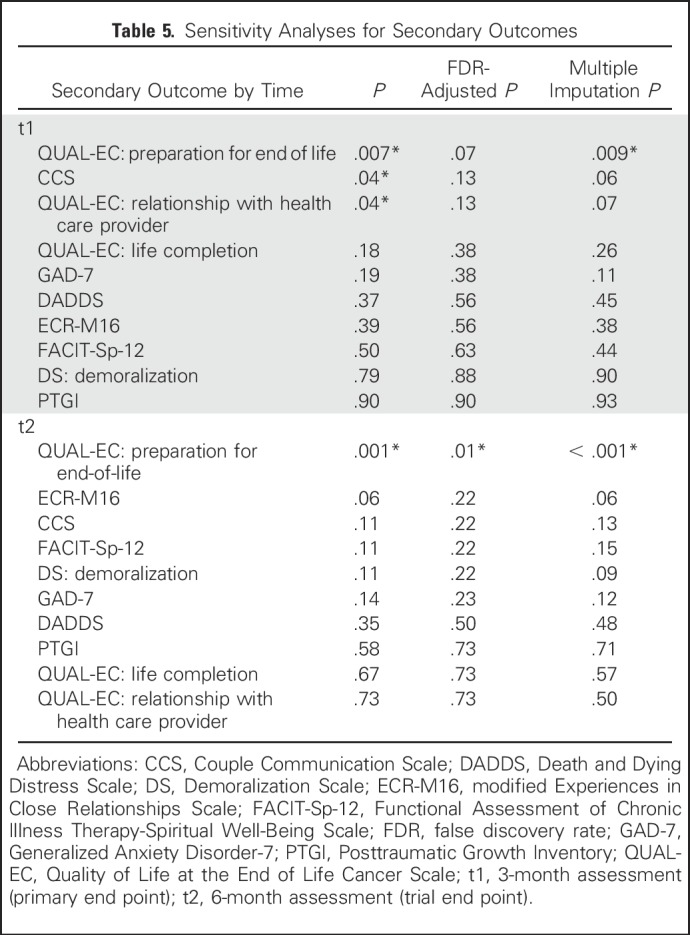
Sensitivity Analyses for Secondary Outcomes

The sample was stratified into low, moderate, and high groups for death anxiety subanalyses. Within each stratum, we tested for treatment effects on secondary outcomes associated with death anxiety.^35,36,56^ CALM participants with moderate death anxiety had significantly lower DADDS scores at both 3 and 6 months than UC participants (*d* = 0.46 and 0.68, respectively). At 6 months, CALM participants also reported less generalized anxiety and demoralization and greater spiritual well-being and attachment security than UC participants in the same DADDS range (*d* range, 0.43 to 0.50; Table 6). No other effects were found in the lowest and highest death anxiety strata (Appendix Table A2, online only).

**Table 6.**
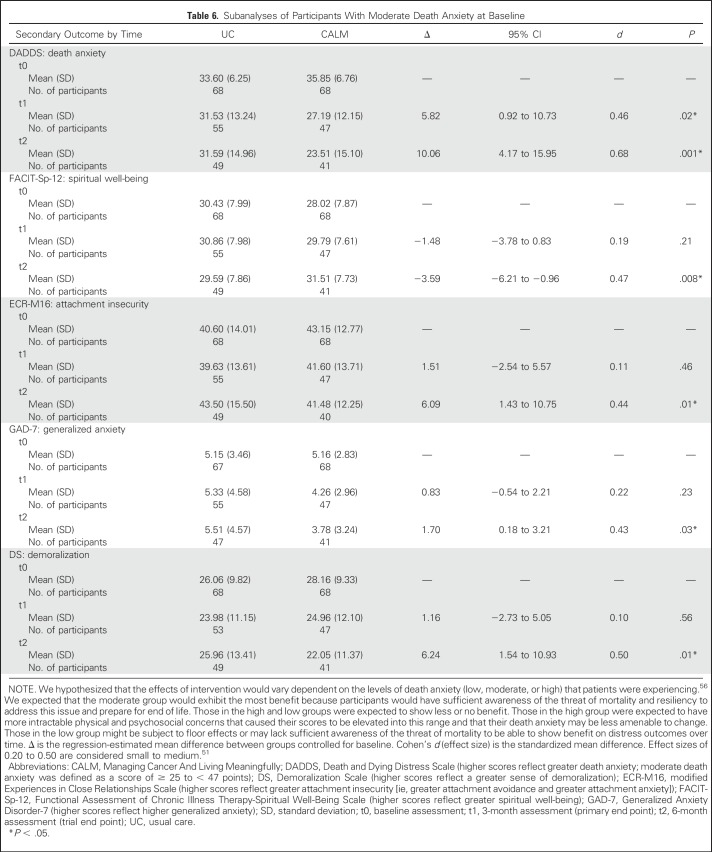
Subanalyses of Participants With Moderate Death Anxiety at Baseline

Results from the mixed-model supplementary analyses supported the main analyses. Appendix Table A3 (online only) lists trial arm × time interactions for all outcomes. The CALM group was expected to show less distress and greater benefit over time relative to the UC group. Significant effects on depressive symptoms, preparation for end of life, and CCS were found. Appendix Table A4 (online only) lists the mixed-model estimated means that explain the significant trial arm × time interactions. The CALM group showed a pattern of steeper decline in depressive symptoms and greater end-of-life preparation over time than the UC group. With regard to the CCS, the two groups seemed to differ at baseline and came to parity over time.

## DISCUSSION

In this RCT of a tailored supportive-expressive therapy for patients with advanced disease and expected prognosis of at least 12 months,^22^ we found significant improvement in the intervention group in the severity of depressive symptoms at 3 months compared with UC, with an apparently greater effect at 6 months compared with UC. CALM was effective in achieving clinically important reductions in depressive symptom severity at 3 and 6 months in participants with at least threshold symptoms and in the rate of remission of threshold symptoms by trial end. We also found a significant treatment effect that favored CALM at the 6-month end point for greater end-of-life preparation compared with UC.

Among participants who were not depressed at baseline, those who received CALM were less likely to report threshold symptoms at the primary end point, which suggests that CALM may help to prevent the onset of depressive symptoms that may otherwise grow over time in individuals with advanced disease.^55^ Although some have suggested the restriction of depression intervention trials in cancer to participants with major depression to avoid floor effects,^57^ this approach may obscure effects on prevention of depressive symptoms in patients without depression.

Evidence with regard to the mechanisms by which CALM exerts its effects will be reported in a separate publication. These mechanisms may include the opportunity for participants to discuss communication with health care providers, to address the effect of their disease on their self-concept and family relationships, to find or reclaim a sense of meaning and purpose in life, to express and manage fears and wishes related to the end of life, and to begin preparations for end of life. CALM addresses these concerns to alter what we have termed a final common pathway of distress that leads to depression in this population.^21^ Such a targeted approach is consistent with the view that positive outcomes and sustained improvement are most likely to occur when the treatment of depression is directed at etiologic and pathogenic factors^58^ and at subsystems of variables that interact in specific contexts.^59^

The study findings suggest that participants with moderate levels of distress about dying and death benefited most from CALM therapy in terms of reduction of such distress and improvement on the secondary outcomes of generalized anxiety, demoralization, spiritual well-being, and attachment security. Those with the lowest levels of death-related distress may be managing death-related concerns effectively and/or may be nonreflective about them; those with the highest levels may feel too overwhelmed to be able or willing to participate in conversations about such issues.^56^ Additional research is needed to clarify which patients might benefit most from CALM and to identify the optimal point in the disease trajectory for CALM to be initiated.

Limitations of this study include that it was conducted at a single site in a large Canadian city with primarily English-speaking, white, well-educated participants, who may not be representative of other settings. The recruitment rate from oncology clinics is comparable to that with other psychotherapeutic interventions in similar settings,^60^ although this may limit the generalizability of the findings. Strengths include the relatively high intervention compliance and completion rates. More than 77% of participants randomly assigned to CALM were compliant with the intervention, and only 10% of those withdrew or were lost to follow-up over 6 months, mainly as a result of disease progression.

In summary, the findings of this RCT suggest that CALM therapy may help to relieve and prevent depressive symptoms in individuals with advanced disease and help patients to address preparations for the end of life. Additional research is needed to explore the optimal timing of CALM, the specific mechanisms of therapeutic action, the most appropriate and meaningful outcome measures, and feasibility and effectiveness of CALM in diverse cultural and clinical settings.

## Figures and Tables

**Table A1.**
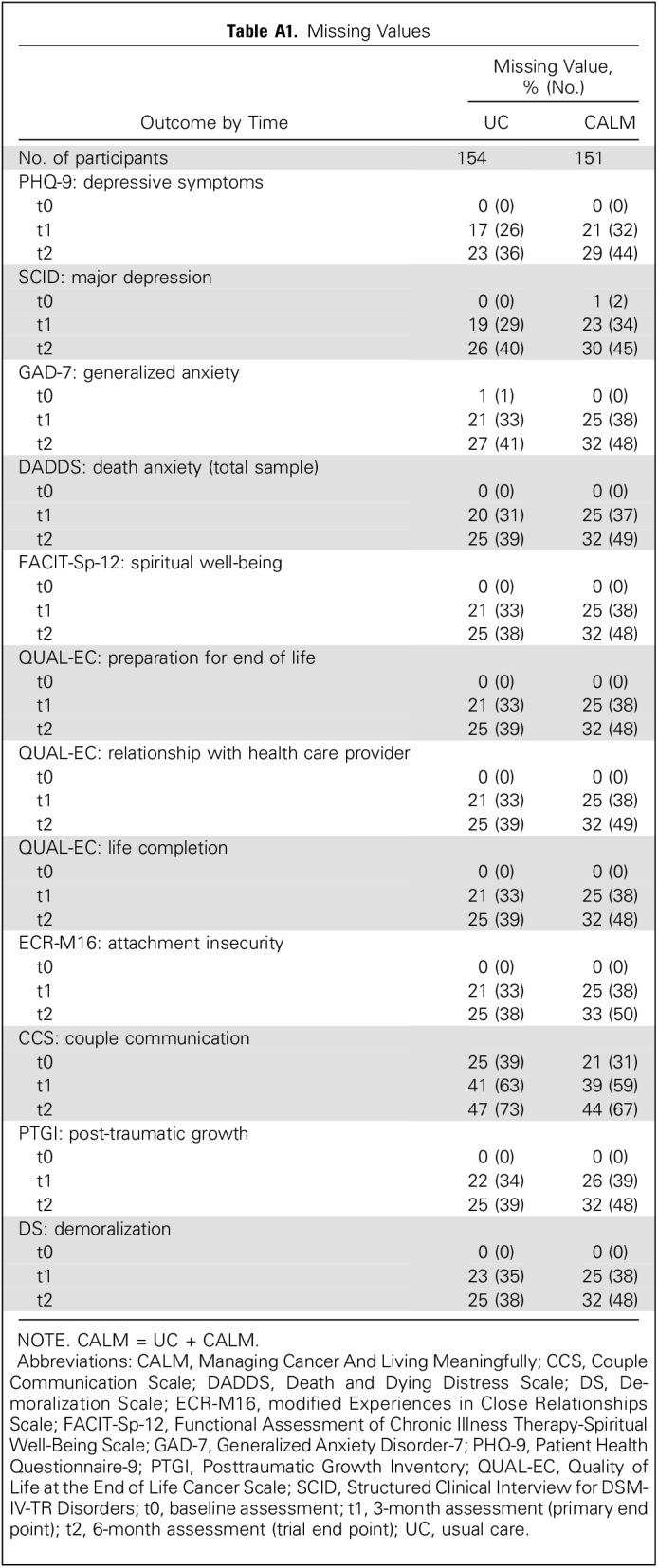
Missing Values

**Table A2.**
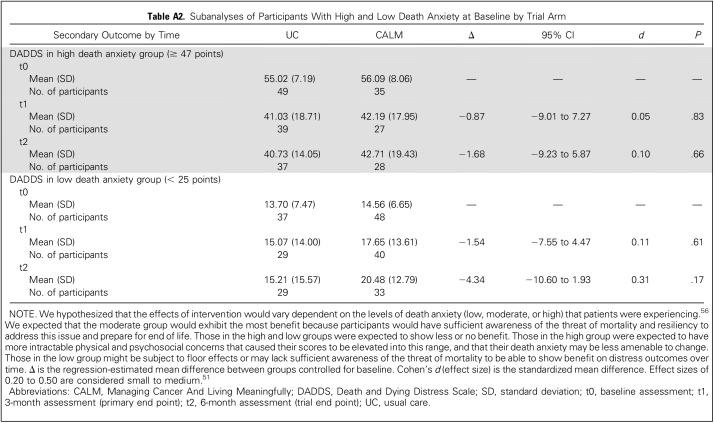
Subanalyses of Participants With High and Low Death Anxiety at Baseline by Trial Arm

**Table A3.**
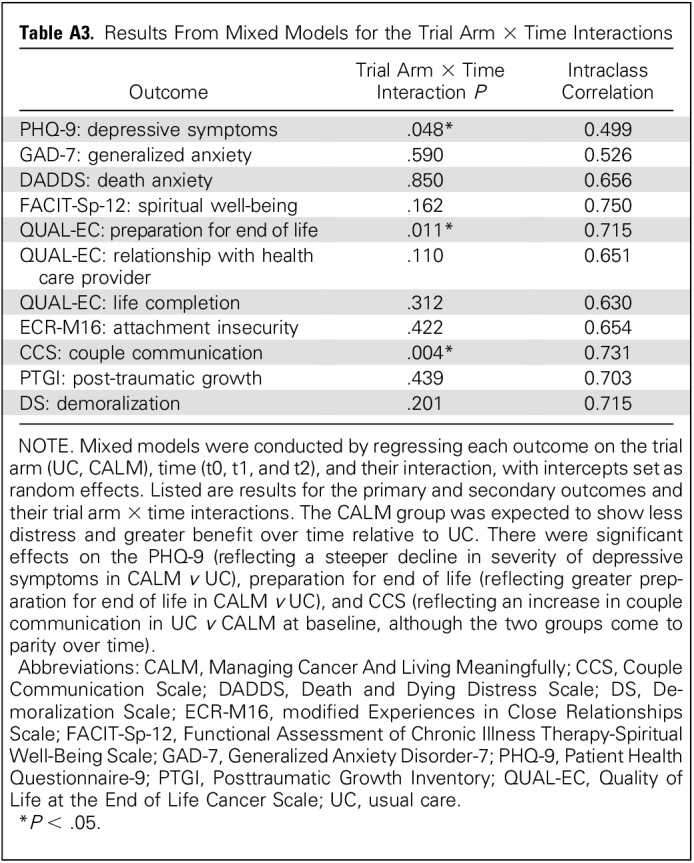
Results From Mixed Models for the Trial Arm × Time Interactions

**Table A4.**
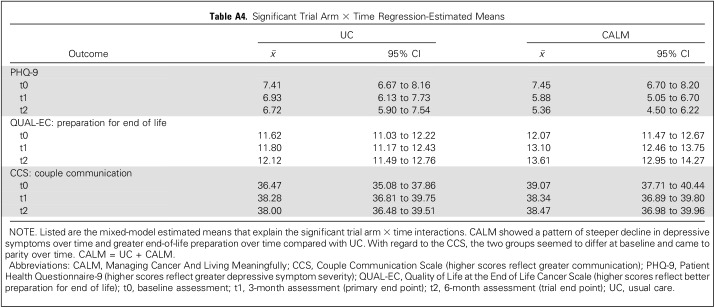
Significant Trial Arm × Time Regression-Estimated Means

## Appendix

### Treatment Integrity Scale: Managing Cancer And Living Meaningfully Evaluation of Therapist Competencies

Therapist:

Case Number:

Case Supervision Dates:

This evaluation is completed on the basis of the case discussion in group supervision and the therapist’s skills as demonstrated in those sessions. Each case presented will have one evaluation form completed. If a skill was not demonstrated in situations that demanded it, then the skill should be rated negatively. If a skill was not demonstrated because it was not applicable, the item can be left blank.

1: Needs improvement 2: Satisfactory 3: Excellent

#### The Therapeutic Relationship

1.___ Shows empathic understanding of patient experiences
2.___ Responds genuinely/honestly to patient thoughts and feelings
3.___ Promotes reflexive awareness (ability to consider multiple psychological responses to an event)
4.___ Acknowledges the realities of the patient’s condition/situation
5.___ Maintains professional boundaries while engaging with patient experiences
6.___ Demonstrates investment/motivation/engagement in the therapeutic process

#### Modulating Affect

7.___ Is able to appropriately modulate the emotional state of the patient
8.___ Demonstrates comfort with emotional distress
9.___ Helps increase patient ability to think about/manage negative emotions/events

#### Shifting Frame

10.___ Shifts among supportive, exploratory, and problem-solving therapeutic frames as necessary
11.___ Adjusts the content and timing of sessions on the basis of the patient’s physical and psychological state

#### Interpretations

12.___ Offers potential explanations for the patient’s pattern of distress, thoughts, or behaviors
13.___ Offers interpretations in the spirit of dialogue and exchange between therapist and patient

Rate the therapist’s skills when addressing each domain, if applicable.

1: Needs improvement 2: Satisfactory 3: Excellent

#### Symptom Management and Communication With Health Care Providers

14.___ Encourages better understanding of disease
15.___ Encourages patient’s active involvement in medical care
16.___ Promotes patient consideration of treatment options
17.___ Supports communication with health care providers

#### Changes in Self and Relations With Close Others

18.___ Explores patient feelings about his/her life history
19.___ Validates patient’s sense of worth in light of his/her accomplishments
20.___ Acknowledges disappointments or regrets that the patient has experienced
21.___ Explores the relational changes imposed by disease
22.___ Explores fears and anxieties about dependency and loss of autonomy
23.___ Encourages appropriate communication and support giving/taking from close others

#### Spirituality or Sense of Meaning and Purpose

24.___ Explores the patient’s spiritual beliefs and/or sense of meaning and purpose in life
25.___ Supports understanding of the personal meaning of their experience of suffering and dying
26.___ Evaluates priorities and goals in the face of advanced disease
27.___ Helps to create new meanings regarding the patient’s life trajectory, goals, and suffering

#### Thinking of the Future, Hope, and Mortality

28.___ Explores patient attitudes towards the future (ie, hopes and fears about living and dying)
29.___ Allows expression of sadness and anxiety about the progression of disease
30.___ Explores feelings about death and dying
31.___ Promotes discussion of advance care planning
32.___ Helps to sustain realistic hope and engagement in life while acknowledging mortality
